# Associations of multiple core stability aspects with drop jump performance

**DOI:** 10.1186/s13102-026-02037-5

**Published:** 2026-09-02

**Authors:** Liang Guo, Meng Wu, Kaiqi Zheng, Xiaokun Gong, Zhongwei Yang, Li Li

**Affiliations:** 1 School of Physical Education & Sports Science, South China Normal University, Guangzhou, Guangdong 510631 People’s Republic of China; 2 Guangzhou Sports Science and Technology Collaborative Innovation Center, Guangzhou Institute of Sports Science, Guangzhou, Guangdong 510500 People’s Republic of China; 3 Department of Health Science and Kinesiology, Georgia Southern University, Statesboro, GA 30458 USA

**Keywords:** Core stability, Drop jump, Lower-extremity, Biomechanics

## Abstract

**Background:**

Drop jump performance (DJ) is essential for many ball sports. Core stability (CS) contributes to trunk control and lower-limb capacity during jump movements. The objective of this study was to investigate the relationships between multiple CS aspects and DJ performance.

**Methods:**

Eighteen male university basketball players were recruited from a university. Kinematic and kinetic data of DJ were collected using infrared cameras and force plates. Five tests—sit-ups (SU), trunk extensor endurance (EE), trunk flexion-extension range of motion (TFE), dominant extremity single-leg stance time (DLS), and dominant extremity single-leg hop distance (DLH)—were used to assess core strength, endurance, flexibility, motor control, and functionality, respectively. Pearson correlation and multiple linear regression assessed the relationship between CS and DJ performance.

**Results:**

DJ height (H_DJ_, *r* = 0.739, *p* < 0.001) and joint moment contribution rate (CR) of ankle (CRankle, *r* = -0.599, *p* = 0.009) were significantly correlated with DLH. Vertical ground reaction force (vGRF, *r* = 0.532, *p* = 0.023), peak joint moment of knee (*r* = 0.494, *p* = 0.037), and CR of hip (CRhip, *r* = -0.554, *p* = 0.017) and knee (CRknee, *r* = 0.514, *p* = 0.029) were significantly correlated with SU. Peak joint moment of knee (PMknee, *r* = 0.611, *p* = 0.007) and ankle (PMankle, *r* = 0.570, *p* = 0.014) were significantly correlated with DLS, and PMankle was also significantly correlated with TFE (*r* = -0.470, *p* = 0.049). Regression models were further explored to explain the H_DJ_ (DLH, R² = 0.546, *p* < 0.001), vGRF (SU, R² = 0.283, *p* = 0.023), CRankle (DLH and TFE, R² = 0. 537, *p* = 0.001 and *p* = 0.029), CRhip (SU, R² = 0.307, *p* = 0.017), CRknee (SU, R² = 0.264, *p* = 0.029), PMknee (DLS, R² = 0.373, *p* = 0.007), and PMankle (DLS, R² = 0.325, *p* = 0.014) by the CS measurements.

**Conclusion:**

Different CS-related characteristics were associated with distinct aspects of DJ performance and lower-extremity biomechanics. Functional capacity was most strongly associated with DJ height, whereas strength- and postural control-related measures were associated with specific biomechanical characteristics. These exploratory findings require confirmation in larger and interventional studies.

## Introduction

The drop jump (DJ) movement is extensively utilized in basketball, volleyball, and other sports requiring explosive jumping ability [1]. Compared with standing vertical jumps, DJ involves greater landing impact and imposes higher mechanical demands on the lower extremities [2]. Previous research has shown that increased landing demands require adjustments in lower-extremity joint moments and their contribution patterns to maintain effective movement execution [3]. These biomechanical adaptations are important for reducing excessive loading during landing [4] and optimizing subsequent take-off performance [5]. The stability and control of the waist-pelvis-hip complex, commonly referred to as core stability (CS), provide an important foundation for coordinating trunk and lower-extremity movements during dynamic tasks [6]. Insufficient CS may alter lower-extremity movement strategies and compromise performance during jumping activities [7].

CS contributes to dynamic movement performance by regulating trunk posture, facilitating force transmission between the trunk and limbs, and supporting coordinated lower-extremity movement [8]. Previous studies have demonstrated that core-related training interventions can modify biomechanical characteristics during jumping tasks. For example, core functional training combined with jump-landing exercises reduced center of pressure displacement during landing and improved vertical jump performance [9]. Similarly, trunk-based core stability training decreased landing-related vertical ground reaction force (vGRF) and peak loading rate during DJ [10], while integrated core training reduced excessive knee joint internal rotation during landing [11]. However, most previous studies have examined CS through comprehensive training programs incorporating multiple core components [10], making it difficult to determine the specific contribution of individual CS characteristics to DJ performance and lower-extremity biomechanics.

CS is a multidimensional construct involving strength, endurance, flexibility, movement control, and functionality [12]. Different core exercises may selectively influence distinct aspects of CS and subsequently modify movement strategies during dynamic tasks [13]. Previous studies have attempted to evaluate individual CS characteristics using specific tests. For example, Blaiser et al. [14] investigated multiple CS-related assessments, including core strength, endurance, neuromuscular control, proprioception, and functional performance, in relation to lower-extremity injury risk. Liang et al. [15] further categorized CS into five components and assessed strength (sit-ups, SU), endurance (trunk extensor endurance, EE), flexibility (trunk flexion-extension range of motion, TFE), movement control (dominant extremity single-leg stance, DLS), and functionality (dominant extremity single-leg hop distance, DLH). Their findings indicated that different CS components showed different relationships with countermovement jump performance [16]. However, whether these distinct CS aspects are differentially associated with DJ performance and the accompanying lower-extremity biomechanical characteristics remains unclear.

Therefore, this study aimed to investigate the associations between five aspects of CS and DJ performance, including jump height and lower-extremity biomechanical characteristics, in basketball players. It was hypothesized that the five aspects of CS would show distinct associations with different characteristics of DJ performance. Specifically, functional CS (represented by DLH) is expected to show a positive relationship with DJ height, while core strength (SU), motor control (DLS), and flexibility (TFE) are expected to be significantly associated with lower-extremity biomechanical measurements during DJ.

## Methods

### Participants and testers

A sample of eighteen university amateur male basketball players was recruited using flyers and in-class announcements from a university. Regular basketball practice (more than 3 times per week for longer than 1 h each time) in the past 2 years was required to participate. Anyone with lower back or lower-extremity injuries or disease within the previous year was excluded from participation. To determine the dominant leg, participants were asked, “Which foot kicks the ball farther when you kick with maximum force?” All 18 participants were identified as having a left dominant leg. The average height was (1.74 ± 0.02) m, the average body mass was (65.6 ± 1.6) Kg, and the average age was (21.9 ± 0.5) years. This study was approved by the university Ethics Committee, and all participants signed informed consent forms before the tests. All tests in this study were conducted by an expert researcher and three graduate students.

### Sample size assessment

The initial sample size was calculated based on a correlation analysis. Based on data from a previous related study, the correlation coefficient between hip isometric strength and knee joint moment during landing was “-0.42” [17]. G*Power (Germany) was used to estimate the minimum sample size. The significance level (α) was set at 0.05. Statistical power was set at 0.80. A two-tailed correlation test was used. The minimum sample size calculated was 16. To further justify the adequacy of the sample size for the multiple linear regression analyses, the effect sizes for all seven established prediction models were evaluated. The Cohen’s $\:{f}^{2}$ for the models ranged from 0.359 to 1.203, representing large effects (≥ 0.35).

### Testing procedure

The whole test lasted approximately two hours. First, the participants’ ages, heights, and masses were recorded, and each participant was assigned a code to protect their privacy.

Before testing, participants completed a video-guided 5-minute standardized warm-up protocol developed based on established sports preparation guidelines [18]. The warm-up consisted of 11 dynamic movements, including boxer shuffle, overhead reaching, stretching, high knee marching, torso twists, toe-touch kicks, full torso circles, lateral step toe touches, squats, jumping jacks, and high knees. After testing, participants performed a video-guided 5-minute standardized cool-down protocol consisting of torso twists, rocking side kickers, rocking butt kickers and ventral pulls, arm crossover swings, quadriceps stretching, hamstring stretching, rocking inside thigh stretching, wall chest stretching, and rhomboid stretching [19].

Prior to the formal tests, all participants underwent practice trials to familiarize themselves with the testing procedures. To ensure maximal effort and minimize the potential confounding effects of fatigue, non-endurance tests were placed before endurance tests. After considering the specific requirements of each test comprehensively, the fixed test order was: drop jump (DJ), trunk flexion-extension range of motion (TFE), dominant extremity single-leg stance time (DLS), dominant extremity single-leg hop distance (DLH), sit-ups (SU), and trunk extensor endurance (EE). Sufficient rest was provided between tests and between trials to prevent fatigue. For all tests, the best trial from the successful attempts was retained for subsequent analysis.

### Drop Jump (DJ) test

After equipment calibration, participants stood on a box, feet shoulder-width apart, and dropped forward smoothly without horizontal velocity. A box height of 45 cm was selected based on the age and male gender of the participants, consistent with established methodologies for amateur athletes [2]. The box was placed 10 cm from the force plates. After landing, participants performed a maximum vertical jump with arm swing, ensuring each foot landed on a separate force plate. They were instructed to jump as if attempting a basketball rebound. Three successful trials were recorded for each participant, with at least 30 s of rest (or longer if necessary) between trials to ensure maximal effort. The trial that produced the greatest jump height (best trial) was retained for subsequent statistical analyses.

### Data collection and processing

**Fig. 1:**
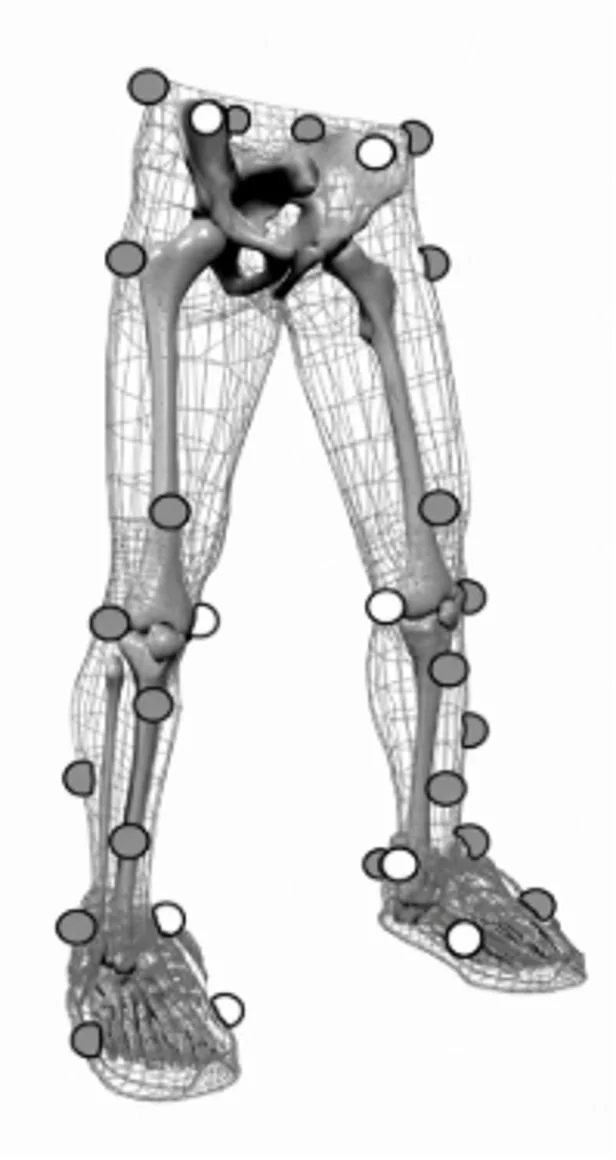
Marker locations used to define the hip, knee, and ankle joints. The white markers, including the left and right anterior superior iliac spine, bilateral medial femoral condyle, bilateral medial malleoli, and bilateral fifth metatarsal heads markers, were removed after static capturing

All participants wore identical shoes to prevent variability from different footwear during the DJ test. Motion data during the DJ test were recorded using a Vicon motion capture system (Vicon Metrics Ltd., Oxford, UK) with eight infrared cameras, set at a sampling frequency of 200 Hz. Thirty markers (14.0 mm in diameter) were attached to participants’ lower extremities based on a lower-extremity model to identify the hip, knee, and ankle joints (Fig. 1) [20]. The white markers were removed after static capture (Fig. 1). Ground reaction force (GRF) data were collected synchronously using two 90 × 60 cm force plates (AMTI, Watertown, USA) at a sampling frequency of 1000 Hz, with five force plate data sets corresponding to one video data set. Before the test, the Vicon motion capture system and force plates were properly calibrated and prepared, including static and dynamic calibration.

The data collected by the Vicon motion capture system were imported into Visual 3D software (C-Motion Inc., Germantown, USA) for further processing and analysis. Kinematic and kinetic data were filtered using zero-lag Butterworth low-pass filters with cut-off frequencies of 44 Hz and 14 Hz, respectively. The cutoff frequencies were determined based on residual analysis to minimize signal noise without distorting the true biomechanical signals, following standard biomechanical data processing recommendations [21]. The DJ was defined as the complete support phase, beginning with the initial ground contact of the dominant leg toe and ending at take-off. The initial ground contact and take-off were identified from force plate data using a vGRF threshold of 10 N. Lower-extremity biomechanics during the DJ were assessed using peak joint moments (PMs) and moment contribution rates (CRs) at the hip, knee, and ankle, together with peak vertical ground reaction force (vGRF). All PM and vGRF variables were normalized to body mass for subsequent analyses.

The contribution rate (CR) for the hip joint was calculated as:

$$\mathrm{CRhip}=\mathrm{PMhip}\:/\:\left(\mathrm{PMhip}+\mathrm{PMknee}+\mathrm{PMankle}\right),$$

with similar formulas applied for the knee and ankle CR. Net Joint internal moments in the sagittal plane were used to evaluate force generation in the lower-extremity joints, computed through inverse dynamics based on force plate and kinematic data. The maximum vertical displacement of the left anterior superior iliac spine (ASIS) marker was used as an estimate of maximum DJ height (H_DJ_), calculated as:

$$\mathrm{H}_{\mathrm{DJ}}=\mathrm{Hmax}-\mathrm{Hbox}+0.45,$$

where Hmax is the maximum vertical height of the iliac spine marker, Hbox is the height when participants were on the box, and 0.45 m is the box height.

### Core Stability (CS) related tests

Five tests were selected to evaluate different aspects of CS based on the previous studies [1, 12] : TFE for flexibility; DLS for motor control; DLH for function; SU and EE for strength and endurance. 

#### Trunk Flexion-Extension range of motion (TFE) test

**Fig. 2:**
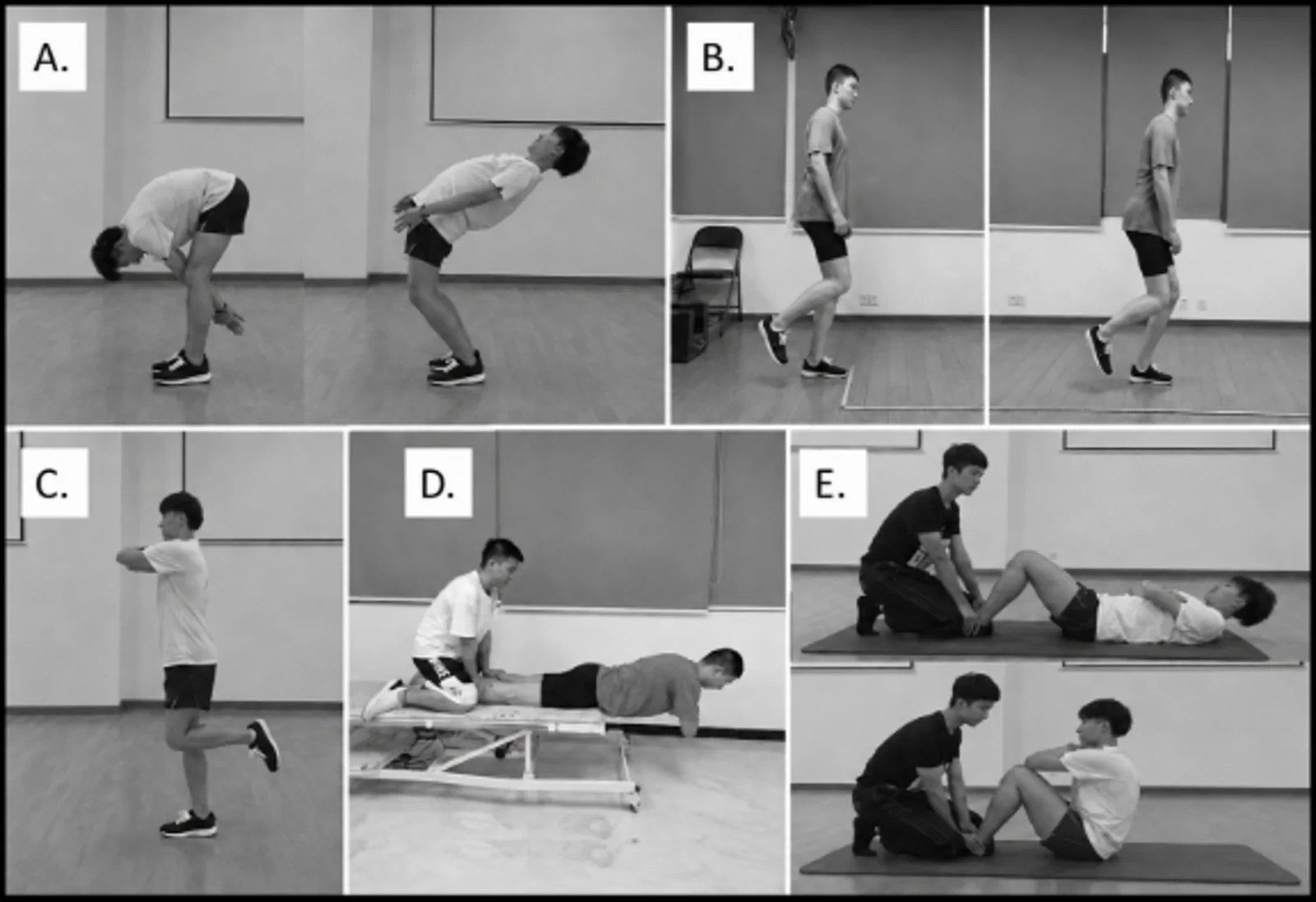
Five CS-related measurements. **A** From left to right: trunk flexion (TF), neutral, and extension (TE) range of motion test, where trunk flexion to extension range of motion (TFE) was recorded as the sum of TF and TE. **B** Dominant extremity single-leg hop distance (DLH) test. **C** Dominant extremity single-leg stance (DLS) test. **D** Trunk extensor endurance (EE) test, and **E** Sit-ups (SU) test

Measurements were taken of the distance between cervical vertebra 7 (C7) and sacral vertebra 1 (S1) while participants stood barefoot in a neutral position. These landmarks were identified and marked. Participants then flexed forward as far as possible while keeping their pelvis stabilized, following the instruction, " hunch forward as far as you can, keeping your hips still.” The distance between C7 and S1 was remeasured, and the difference between the neutral and flexed positions was recorded as the trunk flexion range of motion (TF). For trunk extension, participants extended backward as far as possible, and the distance was remeasured, with the difference recorded as the trunk extension range of motion (TE). The total trunk flexion and extension (TFE) was calculated as the sum of TF and TE. All measurements were recorded in centimeters to the nearest tenth. Refer to Fig. 2A for details.

#### Dominant extremity single-leg hop distance (DLH) test

In this test, participants hopped and landed on their dominant leg, maintaining their landing for at least 2 s for a successful trial (Fig. 2B). The distance was marked with tape and measured from toe to toe. Each participant performed three hops, with the longest distance recorded in centimeters to the nearest tenth and subsequently normalized to body height for analysis. The requirement to achieve a controlled single-leg landing and maintain postural stability incorporates dynamic balance and functional core stability demands. Therefore, the DLH test was considered a functional assessment reflecting the combined contribution of lower-extremity power and dynamic core-related postural control.

#### Dominant extremity single-leg stance (DLS) test

Wearing sports shoes, participants stood on a hard laboratory floor with their arms crossed over their chest and eyes closed. They then lifted their non-dominant leg, maintaining a single-leg stance on their dominant leg with the raised knee flexed to 90° and the hips aligned, ensuring no contact between the legs. This test was repeated three times, with the best of three trials recorded (Fig. 2C). The time began when participants lifted their non-dominant foot off the ground and ended when one of the following occurred: (1) uncrossing of arms, (2) touching the floor with the raised foot, (3) moving the stance foot, (4) reaching a maximum of 45 s, or (5) opening their eyes. Time was recorded in seconds.

#### Sit-Ups (SU) test

Participants began this test in a hook-lying position, with knees flexed at 90°, arms across the chest, and each hand placed on the opposite shoulder. Feet were secured by a partner. A complete sit-up required the scapulae to touch the mat in the lying position and elbows to touch the knees in the sitting position (Fig. 2E). Participants performed as many sit-ups as possible within 20 s. Verbal cues and motivational statements were provided by the primary tester every five seconds. The number of repetitions was manually counted by the primary tester, and the entire test was video-recorded for subsequent verification to ensure counting accuracy.

#### Trunk Extensor Endurance (EE) test

Participants performed this test while lying prone on a medical bed, with the anterior superior iliac spines at the edge of the bed to support their body weight. The pelvis, hips, and knees were secured by a tester. When instructed, participants assumed a horizontal position, with arms crossed over their chest and elbows pointed downward (Fig. 2D). This position was held for as long as possible. Verbal cues and motivational statements were provided by the tester every 10 s. The test ended when the participant could no longer maintain a horizontal position or if the elbows touched the floor. Time was recorded in seconds.

### Statistical analysis

All results were analyzed using SPSS for Windows (version 23.0, IBM Corporation, Armonk, NY, USA). Normality was assessed with the Kolmogorov–Smirnov test. If data were not normally distributed, a log transformation was applied, followed by reassessment of normality. Pearson correlation coefficients were calculated to examine the associations between CS-related measures and DJ variables. The 95% CIs for Pearson correlation coefficients were estimated using percentile bootstrap resampling with 5,000 samples. Stepwise multiple linear regression was conducted to explore the variance in jump height and lower-extremity biomechanics explained by CS-related measurements. Jump height and lower-extremity biomechanical variables from the DJ tests were entered as dependent variables, and CS-related measurements were independent variables. The use probabilities of F were set as 0.05 and 0.10 for entering and deleting, respectively. Because this study was exploratory and hypothesis-generating, no formal adjustment for multiple comparisons was applied. The significance level was set at *p* ≤ 0.05 for all tests.

## Results

Table 1 summarizes participants’ basic information and data from core stability (CS)-related tests.

**Table 1:** The basic information of participants and data of CS-related measurements

	Mean	SD
Age (year)	21.9	2.11
Body Height (m)	1.74	0.07
Body Mass (kg)	65.6	6.92
DLH	1.16	0.10
SU (#)	18.6	2.17
DLS (s)	36.4	7.34
TFE (cm)	18.2	3.34
EE (s)	102.8	29.1

*DLH* Dominant extremity single-leg hop distance normalized to body height, *SU* Sit-ups in 20 s, *DLS* Dominant extremity single-leg stance time, *TFE* Trunk flexion and extension range of motion, *EE* Trunk extensor endurance

Table 2 presents DJ height and lower-extremity biomechanical data.

**Table 2:** The DJ height and lower-extremity biomechanical data

	Mean	SD
PMhip (Nm/kg)	2.28	0.44
PMknee (Nm/kg)	2.17	0.51
PMankle (Nm/kg)	1.66	0.26
CRhip	0.37	0.04
CRknee	0.35	0.05
CRankle	0.27	0.03
vGRF (N/kg)	16.0	3.15
H_DJ_ (m)	0.55	0.05

*PMhip* Peak moment of hip extension normalized to body mass, *PMknee* Peak moment of knee extension normalized to body mass, *PMankle* Peak moment of ankle plantarflexion normalized to body mass, *CRhip* Contribution rate of hip moment, *CRknee* Contribution rate of knee moment, *CRankle* Contribution rate of ankle moment, *vGRF* Peak vertical ground reaction force normalized to body mass, *H*_*DJ*_ Maximum height during drop jump

Table 3 shows the relationships between CS-related measurements and DJ performance. DLH had a significant positive correlation with jump height during DJ (H_DJ_, *r* = 0.739, *p* < 0.001) and a significant negative correlation with contribution rate of ankle moment (CRankle, *r* = -0.599, *p* = 0.009). Sit-ups (SU) were significantly correlated with peak moment of knee extension (PMknee, *r* = 0.494, *p* = 0.037), contribution rate of knee moment (CRknee, *r* = 0.514, *p* = 0.029), and vGRF (*r* = 0.532, *p* = 0.023), and showed a significant negative correlation with contribution rate of hip moment (CRhip, *r* = -0.554, *p* = 0.017). Dominant extremity single-leg stance time (DLS) was significantly correlated with PMknee (*r* = 0.611, *p* = 0.007) and peak moment of ankle plantarflexion (PMankle) (*r* = 0.570, *p* = 0.014). Trunk flexion and extension range of motion (TFE) was significantly negatively correlated with PMankle (*r* = -0.470, *p* = 0.049).

**Table 3:** The relationships between CS-related measurements with DJ height and lower-extremity biomechanical variables

		PMhip	PMknee	PMankle	CRhip	CRknee	CRankle	vGRF	H_DJ_
DLH	r	0.446	0.440	-0.008	0.146	0.235	-0.599##	0.456	0.739##
	95%CI	[0.120, 0.702]	[-0.075, 0.753]	[-0.452, 0.460]	[-0.241, 0.543]	[-0.277, 0.601]	[-0.833, -0.288]	[0.091, 0.715]	[0.491, 0.897]
	p	0.064	0.068	0.974	0.565	0.347	0.009	0.057	< 0.001
SU	r	-0.162	0.494#	0.206	-0.554#	0.514#	-0.027	0.532#	0.161
	95%CI	[-0.607, 0.224]	[-0.075, 0.781]	[-0.360, 0.522]	[-0.804, -0.196]	[0.093, 0.841]	[-0.349, 0.427]	[0.070, 0.786]	[-0.463, 0.613]
	p	0.522	0.037	0.412	0.017	0.029	0.915	0.023	0.523
DLS	r	0.436	0.611##	0.570#	-0.158	0.267	-0.206	0.446	0.453
	95%CI	[0.006, 0.759]	[0.243, 0.845]	[0.208, 0.805]	[-0.570, 0.383]	[-0.210, 0.651]	[-0.634, 0.262]	[0.028, 0.757]	[-0.051, 0.786]
	p	0.070	0.007	0.014	0.532	0.284	0.413	0.064	0.059
TFE	r	-0.216	-0.224	-0.470#	0.054	0.044	-0.151	-0.172	-0.362
	95%CI	[-0.582, 0.289]	[-0.644, 0.285]	[-0.809, 0.010]	[-0.370, 0.467]	[-0.429, 0.465]	[-0.584, 0.327]	[-0.507, 0.151]	[-0.739, 0.238]
	p	0.389	0.372	0.049	0.831	0.863	0.550	0.495	0.140
EE	r	-0.190	0.133	0.089	-0.293	0.190	0.119	-0.092	-0.244
	95%CI	[-0.580, 0.236]	[-0.257, 0.480]	[-0.369, 0.455]	[-0.627, 0.079]	[-0.281, 0.593]	[-0.269, 0.566]	[-0.480, 0.324]	[-0.701, 0.260]
	p	0.451	0.600	0.725	0.238	0.450	0.637	0.717	0.329

Note: 95% CIs for Pearson correlation coefficients were estimated using bootstrap resampling (5,000 samples). Statistical significance was determined based on the two-tailed Pearson correlation *p*-values

#: *p* < 0.05, ##: *p* < 0.01

**Fig. 3:**
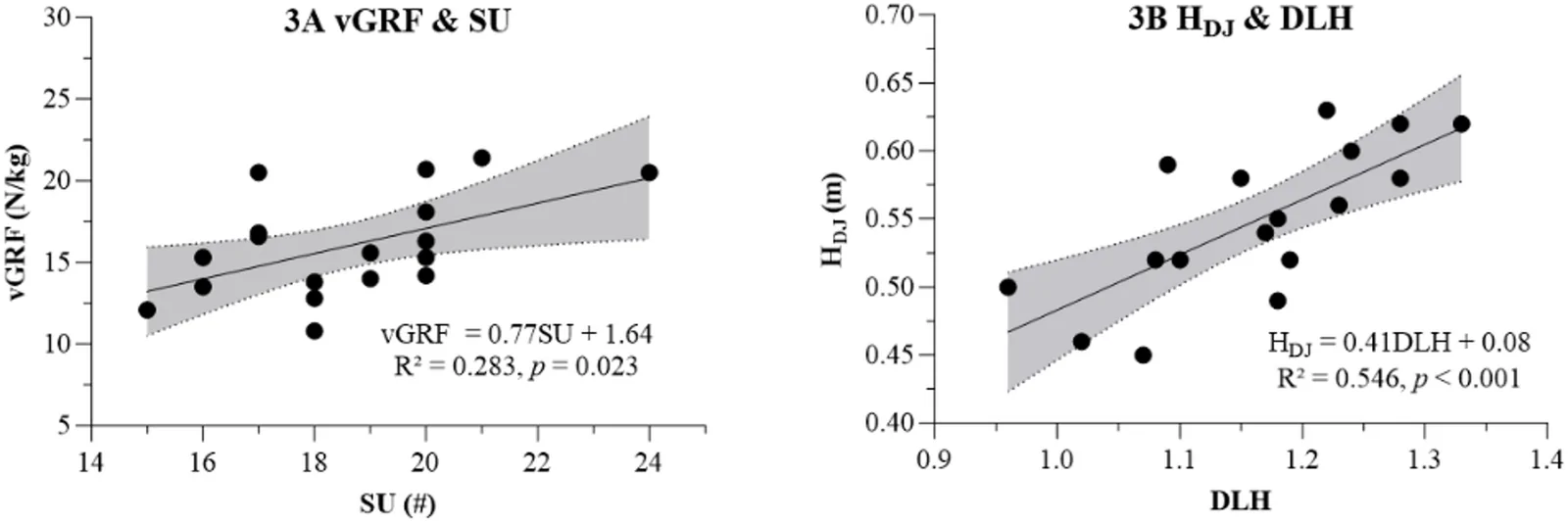
Trend lines with 95% CI of linear regression analyses of the vertical ground reaction force (vGRF, R² = 0.283, *p* = 0.023, 3 **A**) and jump height during DJ (H_DJ_, R² = 0.546, *p* < 0.001, 3**B**) with the 5 CS-related measurements which included dominant extremity single-leg hop distance (DLH), sit-ups (SU), dominant extremity single-leg stance time (DLS), trunk flexion and extension range of motion (TFE), and trunk extensor endurance (EE)

Figure 3 presents linear regression analyses for vGRF and H_DJ_ using the CS-related measurements. Sit-ups (SU) entered the model for vGRF, explaining 28.3% of the variance (adjusted R² = 0.238, B = 0.773, SE = 0.307, 95% CI [0.121, 1.424], *p* = 0.023). DLH entered the H_DJ_ model, explaining 54.6% of the variance (adjusted R² = 0.517, B = 0.406, SE = 0.093, 95% CI [0.210, 0.603], *p* < 0.001). Variance inflation factors (VIFs) for both models were 1.000, indicating a low risk of collinearity.

**Fig. 4:**
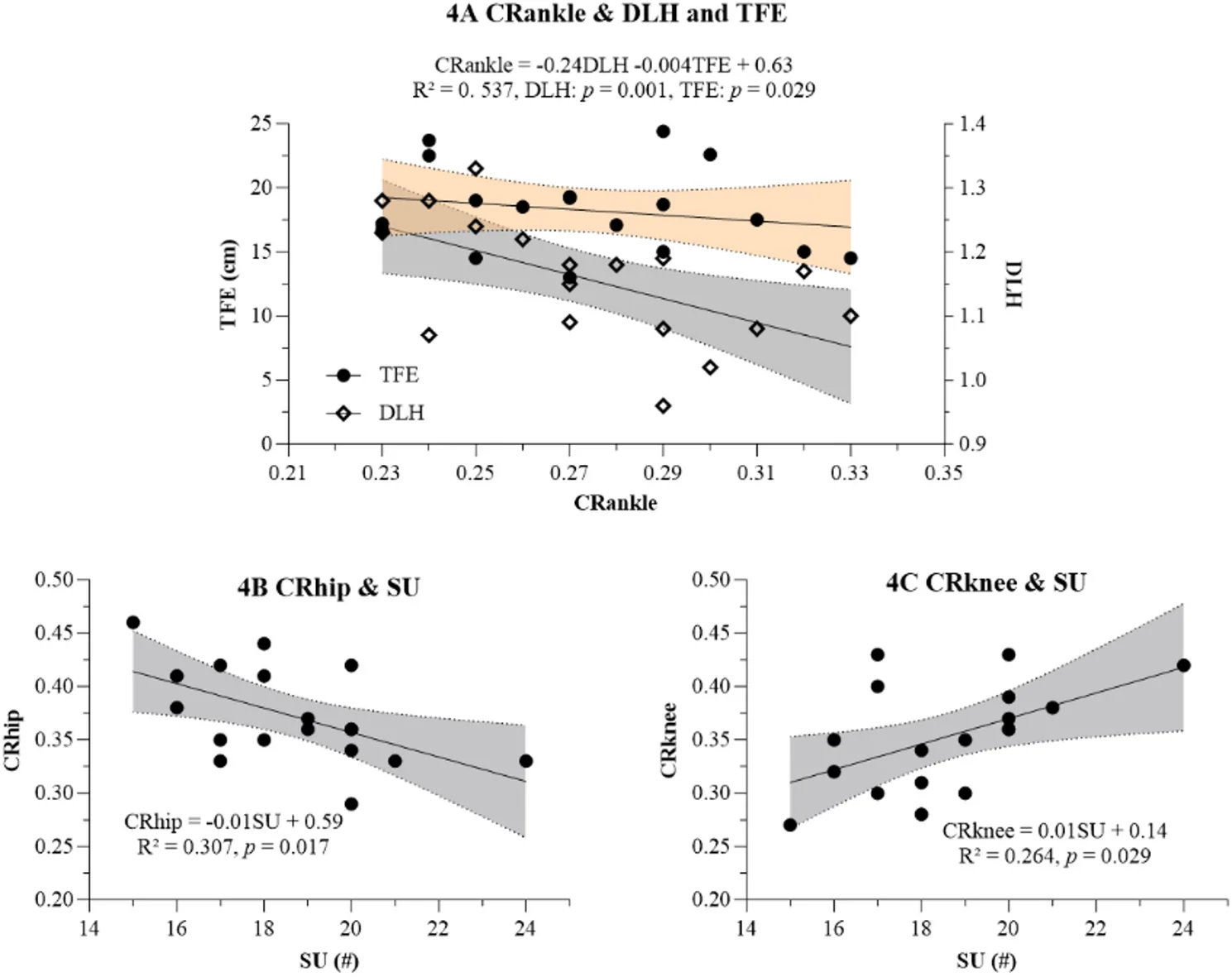
Trend lines with 95% CI of linear regression analyses of the contribution rate of hip moment (CRhip, R² = 0.307, *p* = 0.017, 4**B**), contribution rate of knee moment (CRknee, R² = 0.264, *p* = 0.029, 4 **C**), and contribution rate of ankle moment (CRankle, R² = 0. 537, DLH: *p* = 0.001, TFE: *p* = 0.029, 4 **A**) with the 5 CS-related measurements, which included dominant extremity single-leg hop distance (DLH), sit-ups (SU), dominant extremity single-leg stance time (DLS), trunk flexion and extension range of motion (TFE), and trunk extensor endurance (EE)

Figure 4 shows linear regression analyses of the contribution rates for hip (CRhip), knee (CRknee), and ankle (CRankle) moments using the five CS-related measurements. DLH and TFE entered the CRankle model, explaining 53.7% of the variance (adjusted R² = 0.475; for DLH: B = -0.242, SE = 0.059, 95% CI [-0.368, -0.116], *p* = 0.001; for TFE: B = -0.004, SE = 0.002, 95% CI [-0.008, 0.000], *p* = 0.029). SU entered the models for CRhip and CRknee, explaining 30.7% (adjusted R² = 0.264, B = -0.011, SE = 0.004, 95% CI [-0.020, -0.002], *p* = 0.017) and 26.4% (adjusted R² = 0.218, B = 0.012, SE = 0.005, 95% CI [0.001, 0.022], *p* = 0.029) of the variance, respectively. The VIFs for DLH and TFE in the CRankle model were 1.186, while VIFs for SU in the CRhip and CRknee models were 1.000.

**Fig. 5:**
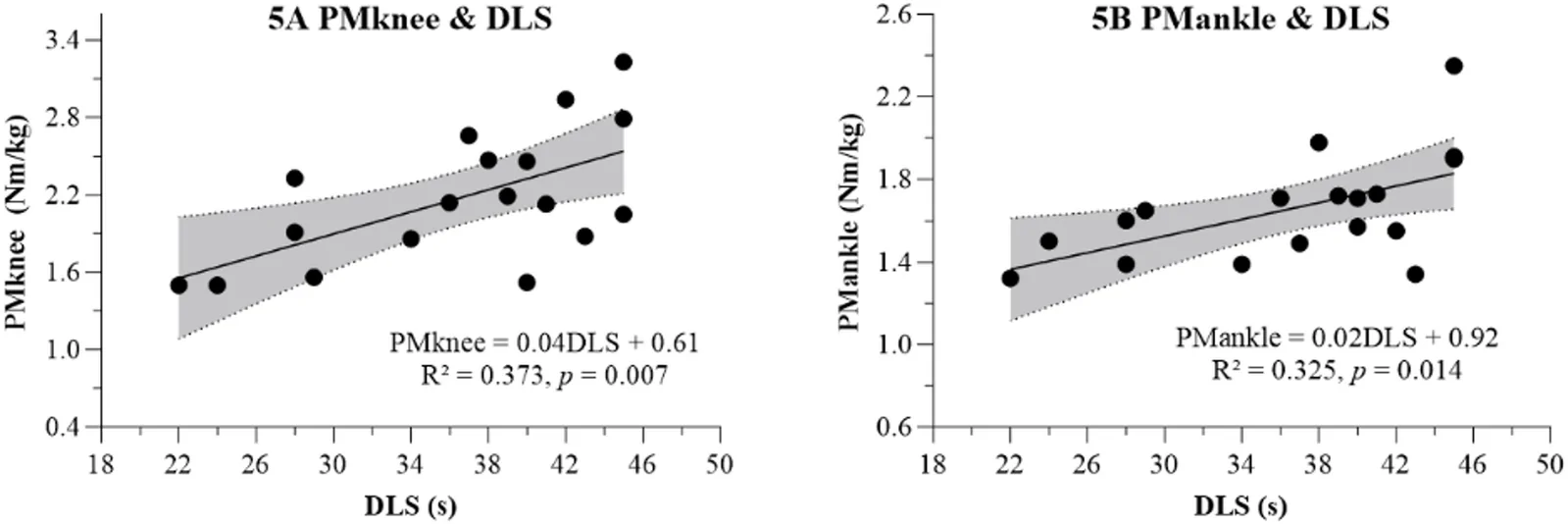
Trend lines with 95% CI of linear regression analyses of the peak moment of knee extension (PMknee, R² = 0.373, *p* = 0.007, 5 **A**) and peak moment of ankle plantarflexion (PMankle, R² = 0.325, *p* = 0.014, 5**B**) with the 5 CS-related measurements, which included dominant extremity single-leg hop distance (DLH), sit-ups (SU), dominant extremity single-leg stance time (DLS), trunk flexion and extension range of motion (TFE), and trunk extensor endurance (EE)

Figure 5 shows linear regression analyses of peak moments for the knee (PMknee) and ankle (PMankle) using the five CS-related measurements. DLS entered the models for PMknee and PMankle, explaining 37.3% (adjusted R² = 0.334, B = 0.043, SE = 0.014, 95% CI [0.013, 0.072], *p* = 0.007) and 32.5% (adjusted R² = 0.283, B = 0.020, SE = 0.007, 95% CI [0.005, 0.036], *p* = 0.014) of the variance, respectively. The VIFs for DLS in both models were 1.000.

Residual diagnostics for all the above regression models confirmed that the assumptions of normality and homoscedasticity were adequately met. 

## Discussion

This study investigated the associations of multiple aspects of core stability (CS) with drop jump (DJ) performance by examining relationships between five CS-related tests, DJ height, and lower-extremity biomechanical variables. The findings demonstrated that different aspects of CS were associated with distinct characteristics of DJ performance. Specifically, dominant extremity single-leg hop distance (DLH) was significantly associated with DJ height (H_DJ_), whereas sit-ups (SU) were significantly associated with vGRF, hip moment contribution rate (CRhip), and knee moment contribution rate (CRknee). Dominant extremity single-leg stance time (DLS) was significantly associated with the peak moments of the knee (PMknee) and ankle (PMankle). Additionally, DLH combined with trunk flexion and extension range of motion (TFE) were significantly associated with ankle moment contribution rate (CRankle). These results suggest that different aspects of CS may be associated with DJ performance through different biomechanical pathways rather than through a uniform mechanism.

Drop jump (DJ) imposes high mechanical demands on the lower extremities, requiring rapid force absorption and generation, wherein core stability (CS) plays a pivotal role in energy transfer [8]. Among the five CS-related assessments, DLH showed the strongest association with H_DJ_, explaining 54.6% of the variance. This result was consistent with previous studies about the relationship between hop distance and vertical jump performance [15, 22]. The relationship may be partly attributed to the functional similarity between DLH and DJ, as both tasks require rapid force generation, postural control, and efficient coordination of lower-extremity segments [23]. During single-leg hopping, mechanical energy must be absorbed and transferred across the hip, knee, and ankle joints to achieve effective propulsion [24]. Therefore, individuals with better DLH performance may demonstrate more effective coordination of proximal and distal segments during explosive movement tasks [25], which may partly explain the observed association with DJ performance. However, DLH should not be interpreted as an isolated measure of core stability. Although the requirement to maintain a single-leg landing position for at least two seconds incorporates elements of functional stability and postural control, hop distance is also strongly influenced by lower-extremity explosive capacity [22]. Therefore, the association between DLH and H_DJ_ may reflect the combined contribution of functional stability, neuromuscular coordination, and lower-limb power.

In contrast to DLH, SU was not significantly associated with H_DJ_ but showed significant associations with vGRF, CRhip, and CRknee. This finding is broadly consistent with previous evidence suggesting that greater core strength does not necessarily translate into improved jump performance [26, 27]. For example, Lago-Fuentes et al. [27] reported no significant improvement in CMJ performance following six weeks of core strength training. Similarly, the present findings suggest that SU performance may be more closely associated with lower-extremity force-generation strategies and inter-joint contribution patterns than with the final jump-height outcome. Specifically, SU was positively associated with CRknee and negatively associated with CRhip, indicating a redistribution of joint contributions during DJ. Greater trunk and pelvic strength may provide a more stable proximal base for force transmission to the lower extremities, thereby influencing how joint moments are distributed across the kinetic chain [8, 16]. Thus, the observed hip–knee contribution pattern may reflect differences in inter-joint coordination rather than superior performance of an individual joint [28].

DLS was significantly associated with PMknee and PMankle. Unlike strength-based tests, DLS reflects single-leg postural stability, integrating proprioceptive feedback, and balance control [29]. Previous studies have shown that stabilization and balance training can improve lower-extremity performance, while proprioceptive function contributes to the regulation of posture and movement during dynamic tasks [30]. The present findings for DLS are consistent with these observations and suggest a specific association between single-leg postural stability and knee and ankle force-generation characteristics during DJ. In contrast, TFE was negatively associated with CRankle and PMankle. Excessive trunk motion may indicate reduced trunk control and altered mechanical energy transmission from the proximal segments to the lower extremities [31]. Such changes may alter the contribution of the ankle joint during force production [32]. These findings highlight that CS-related characteristics may influence specific joint movement patterns even when they do not directly affect jump height.

Interestingly, EE showed no significant associations with H_DJ_ or lower-extremity biomechanical variables. This result may be explained by the specific mechanical demands of DJ. Trunk extensor endurance primarily reflects the ability to maintain force output during prolonged or repetitive activities [1], whereas DJ involves rapid eccentric-concentric force generation within a short time period [33]. Therefore, endurance capacity may have a limited association with the acute mechanical requirements of DJ compared with characteristics related to strength, functional capacity, and neuromuscular control [34]. This finding is broadly consistent with evidence from a previous intervention study [34], suggesting that different CS components may have task-specific relationships with DJ performance.

Although the selected tests have been widely used to assess different aspects of CS [12], these assessments should be interpreted within their specific task characteristics [35]. For example, SU and EE primarily reflect strength and endurance of the core and may be influenced by the contributions from other body segments rather than isolated core muscle capacity. Similarly, DLS and DLH represent integrated functional abilities involving postural control, balance, lower-extremity strength, and neuromuscular coordination. Therefore, the observed associations should be interpreted as relationships between CS-related capability and DJ performance rather than direct effects of individual core components. In addition, the absence of electromyographic measurements limited our ability to examine the underlying neuromuscular activation strategies contributing to the observed biomechanical associations, although such measurements were beyond the primary scope of the present study.

Several additional limitations should be acknowledged. First, the cross-sectional design and relatively small sample size (*n* = 18) limit causal inference and may restrict the generalizability of the findings. Although Pearson correlation and regression analyses provided preliminary insights into the associations between CS-related measures and DJ performance, the multiple statistical comparisons and small sample size may have increased the risks of Type I error and model overfitting, respectively. Therefore, these findings should be interpreted cautiously and require validation in larger samples. Second, although participants completed familiarization trials before testing, potential learning effects across the test battery cannot be completely excluded. The testing order was standardized and sufficient rest intervals were provided to minimize fatigue; however, repeated exposure to the tasks may have influenced performance outcomes. Third, all participants in this study were male basketball players, which limits the generalizability of the findings to female athletes or other populations. Future studies should include female participants and examine whether sex-related differences in neuromuscular characteristics influence the relationship between CS and DJ performance.

Finally, DJ height was determined based on the vertical displacement of the anterior superior iliac spine (ASIS) marker. Marker-based motion capture approaches enable direct measurement of body segment displacement during movement analysis. However, ASIS marker displacement represents the movement of a specific anatomical landmark and may be affected by pelvic rotation and trunk motion during jumping [36]. Therefore, the DJ height reported in this study should be interpreted as ASIS-derived vertical displacement rather than center-of-mass (COM) displacement. Future studies should further validate this approach against established COM-based methods and investigate whether targeted CS interventions can modify DJ performance.

## Conclusion

This study identified a significant positive association between DLH and DJ height. Although SU, DLS, and TFE were not significantly associated with DJ height, they were associated with specific lower-extremity biomechanical characteristics during DJ. These findings suggest that different CS-related capabilities may be associated with distinct aspects of DJ performance in male university basketball players. However, intervention studies are required to determine whether training targeting these characteristics can improve DJ performance.

## Data Availability

The datasets supporting the conclusions of this study are available from the corresponding author on reasonable request.
